# Maximal resection improves survival in MGMT methylated very elderly glioblastoma patients

**DOI:** 10.1007/s11060-025-05417-y

**Published:** 2026-02-09

**Authors:** Dragan Jankovic, Santhosh G. Thavarajasingam, Daniel Scurtu, Manuel V. Baby, Andreas Kramer, Jonathan Weller, Veit Stöcklein, Marc A. Brockmann, Clemens Sommer, Petra Leukel, Marcus Stockinger, Harald Krenzlin, Naureen Keric, Florian Ringel, Darius Kalasauskas

**Affiliations:** 1 Department of Neurosurgery, University Medical Center Mainz, Mainz, Germany; 2 Department of Neurosurgery, LMU University Hospital, 81377 Munich, Germany; 3 Imperial Brain & Spine Initiative, Imperial College London, London, UK; 4 Faculty of Medicine, Imperial College London, London, UK; 5 Department of Neurosurgery, University Hospital Schleswig-Holstein, Luebeck, Germany; 6 Department of Neuroradiology, University Medical Center Mainz, Mainz, Germany; 7 Institute of Neuropathology, University Medical Center Mainz, Mainz, Germany; 8 Department of Radiooncology and Radiotherapy, University Medical Center Mainz, Mainz, Germany

**Keywords:** Elderly patients, Glioblastoma, MGMT promoter methylation, Surgical resection, Survival

## Abstract

**Background:**

Glioblastoma is the most aggressive primary tumor of the central nervous system, with particularly poor prognosis in elderly patients. Individuals aged ≥ 75 years remain underrepresented in clinical trials, resulting in limited evidence to guide treatment decisions. This study aimed to analyze treatment patterns and survival outcomes in patients aged 75 years or older with newly diagnosed IDH-wildtype glioblastoma.

**Methods:**

We conducted a single-center retrospective study of 108 consecutive patients aged ≥ 75 years who underwent surgical intervention between 2016 and 2022. Patients were stratified by age group (75–79 vs. ≥80 years), surgical modality (biopsy vs. resection), extent of resection (maximal vs. submaximal), MGMT promoter methylation status, and adjuvant therapy. Overall survival (OS) and progression-free survival (PFS) were assessed using Kaplan–Meier analysis and Cox proportional hazards models.

**Results:**

The median age was 79 years, and 46.3% of patients were female. Biopsy alone was performed in 33.3% of patients, while 60.8% underwent surgical resection. Resection significantly improved OS compared with biopsy (*p* = 0.0047), with maximal resection providing superior OS compared with submaximal resection (*p* = 0.00016). The benefit of maximal resection was most pronounced in patients aged 75–79 years and in those with MGMT-methylated tumors. Among adjuvant treatments, radiochemotherapy conferred the strongest survival advantage (HR 0.174, 95% CI 0.086–0.355, *p* < 0.001).

**Conclusions:**

Maximal resection significantly improves survival in glioblastoma patients aged ≥ 75 years, particularly those with MGMT-methylated tumors, and should be considered even in selected patients aged ≥ 80 years. These findings underscore the importance of individualized treatment strategies and support the feasibility of aggressive surgical management in very elderly patients with favorable clinical status.

## Introduction

Glioblastoma is the most aggressive primary tumor of the central nervous system with a median survival ranging from 12 to 15 months [1, 2]. Only 5% of patients survive more than five years [3]. The median age at glioblastoma diagnosis is approximately 64 years, and age-standardized incidence rates continue to rise across all age groups in Western societies. The reported median survival in elderly patients is 5 to 9 months [4–6]. Several reasons have been reported for the poor prognosis in older glioblastoma patients, including reduced treatment intensity, weaker treatment response, increased patient frailty, polypharmacy and potential drug interactions, as well as differences in tumor biology [7–10]. Population-based cancer registry studies reported that elderly patients with glioblastoma less frequently receive multimodal treatment [7, 11]. The treatment of elderly patients with glioblastoma often represents a challenge, as the therapeutic goal must balance prolonging survival with maintaining adequate functional status while limiting the toxicity of chemotherapeutic agents.

Up to date, only three randomized controlled trials (RCTs) have addressed the treatment of elderly patients with glioblastoma [12–14]. The NOA-08 trial compared temozolomide (TMZ) monotherapy with radiotherapy alone in 373 patients with high-grade gliomas and reported no significant difference in overall survival (OS) between these two treatment approaches [12]. In the Nordic trial, patients were randomized into three arms receiving either TMZ, standard radiotherapy with 60 Gy, or short-course hypofractionated radiotherapy (RT) with 34 Gy [13]. No survival differences were observed across the groups. Finally, Perry et al. evaluated hypofractionated RT combined with TMZ versus hypofractionated RT alone, reporting a clear survival benefit in favor of the combined regimen [14]. 

However, despite these pivotal RCTs, there is still a paucity of data specifically addressing the subgroup of very elderly patients, particularly those aged 75 years and above. Most available evidence either excludes such patients or includes them in small numbers, thereby limiting the generalizability of trial results to this age group. In clinical practice, treatment decisions in these patients are often individualized and influenced by performance status, comorbidities and patient preferences [15]. Consequently, the optimal management of glioblastoma in very elderly patients remains uncertain, and real-world data reflecting treatment patterns and outcome in this patient subgroup are scarce.

The aim of this study was to evaluate treatment pattern and survival outcomes in patients aged 75 years or older with histologically confirmed IDH-wildtype glioblastoma.

## Material and methos

### Study design and patient

The single-center, retrospective, observational study reviewed the medical records of consecutive patients aged ≥ 75 years with histologically confirmed IDH-wildtype glioblastoma who underwent surgical intervention, between January 2016 and December 2022. The patient data were de-identified before analysis. All data are reported in accordance with the Strengthening the Reporting of Observational Studies in Epidemiology (STROBE) guidelines for observational studies.

### Eligibility criteria

#### Inclusion criteria

Patients aged ≥ 75 years with histologically confirmed IHD-wildtype glioblastoma who underwent surgical intervention.

#### Exclusion criteria

(1) Patients aged < 75 years; (2) Patients that had IDH-mutant tumors or lacked confirmation of IDH-wildtype status.

### Clinical, radiological, pathological, and treatment parameters

Baseline characteristics included age, sex, functional neurological status at admission and discharge, assessed using the Karnofsky Performance Status (KPS). Radiological features were obtained from pre- and postoperative MRI scans, and molecular tumor profiles from histopathology reports.

Treatment decision was made according to tumor board recommendation. The choice between biopsy-only and surgical resection was determined by a comprehensive assessment of both patient-related and tumor-related factors. Patient-related factors included relevant comorbidities and preoperative functional status, while tumor-related considerations comprised multifocal disease, diffuse or infiltrative growth patterns, and involvement of eloquent or dee-seated brain regions where safe maximal resection was not considered feasible. In cases where the anticipated surgical risk outweighed the potential benefit of resection, a biopsy-only approach was favored to establish histological and molecular diagnosis and to guide further oncological treatment. All patients were treated with either stereotactic biopsy or open surgical resection. A postoperative MRI of the brain performed within 72 h after surgery was used to determine the extent of resection (EOR). EOR was evaluated using the Response Assessment in Neuro-Oncology (RANO) classification for glioblastoma:

- **Maximal resectio**n: complete contrast enhancement (CE) resection (0 cm3 CE + > 5 cm3 nCE) and near total CE resection (≤ 1 cm3 CE);
- **Submaximal resection**: subtotal CE resection (≤ 5 cm3 CE) and partial CE resection (> 5 cm3 CE) [16].

Tumor volume (cm^3^) was segmented manually with the software Brainlab Elements^®^ version 3.1.0. (Brainlab AG, Munich, Germany) using axial, coronal and sagittal contrast-enhanced T1-weighted images. In cases where the tumor was not contrast-enhancing, volumetry was performed using T2-weighted or FLAIR MR images.

Histopathological diagnosis was based on the CNS WHO 2021 classification [17]. To evaluate the MGMT promoter methylation status, tumor tissue samples were preserved as formalin-fixed, paraffin-embedded (FFPE) tissue blocks. DNA was extracted from the FFPE samples using commercially available DNA extraction kits, ensuring high-quality DNA suitable for methylation analysis. Subsequently, bisulfite conversion of the extracted DNA was performed to differentiate between methylated and unmethylated cytosines. Methylation-specific polymerase chain reaction (MSP) was then conducted using primers designed to target methylated and unmethylated regions of the MGMT promoter. For a subset of samples, quantitative analysis was performed using pyrosequencing to determine the methylation level at specific CpG sites within the MGMT promoter region.

Postoperatively, patients received adjuvant therapy, which was categorized as chemotherapy alone, radiotherapy alone, or combined radiochemotherapy. Patients who did not receive any tumor-specific treatment following histopathological confirmation of glioblastoma were classified as “best supportive care”.

The primary outcome was overall survival (OS), defined as the time from initial diagnosis until the patient´s death. The secondary outcome was progression-free survival (PFS), defined as the interval from surgery to radiologically confirmed progression.

### Statistical analysis

Statistical analyses were conducted using R software (version 4.5.0, R Foundation for Statistical Computing, Vienna, Austria). Overall survival (OS) and progression-free survival (PFS) were evaluated using Kaplan–Meier methods, with curves generated for groups stratified by age (75–79 years vs. ≥80 years), surgical approach (biopsy vs. resection), pre-operative Karnofsky Performance Status score, MGMT promoter methylation status, type of tumour-specific therapy received, and extent of resection (maximal vs. sub-maximal). Univariate and multivariate Cox proportional hazards models were applied to estimate hazard ratios (HRs) for each factor. An HR < 1.00 was interpreted as an association with improved OS or PFS, whereas an HR > 1.00 indicated an association with worse survival outcomes. Box plots were used to visualize changes in KPS from admission across these prognostic categories. Stacked bar charts illustrated the distribution of surgical interventions (resection vs. biopsy) and the distribution of tumor-specific therapy modalities within the cohort. All analyses were performed using standard R packages, and results are reported with the corresponding HRs and survival curves for each stratification variable [18]. 

## Results

### Patients demographics

**Fig. 1:**
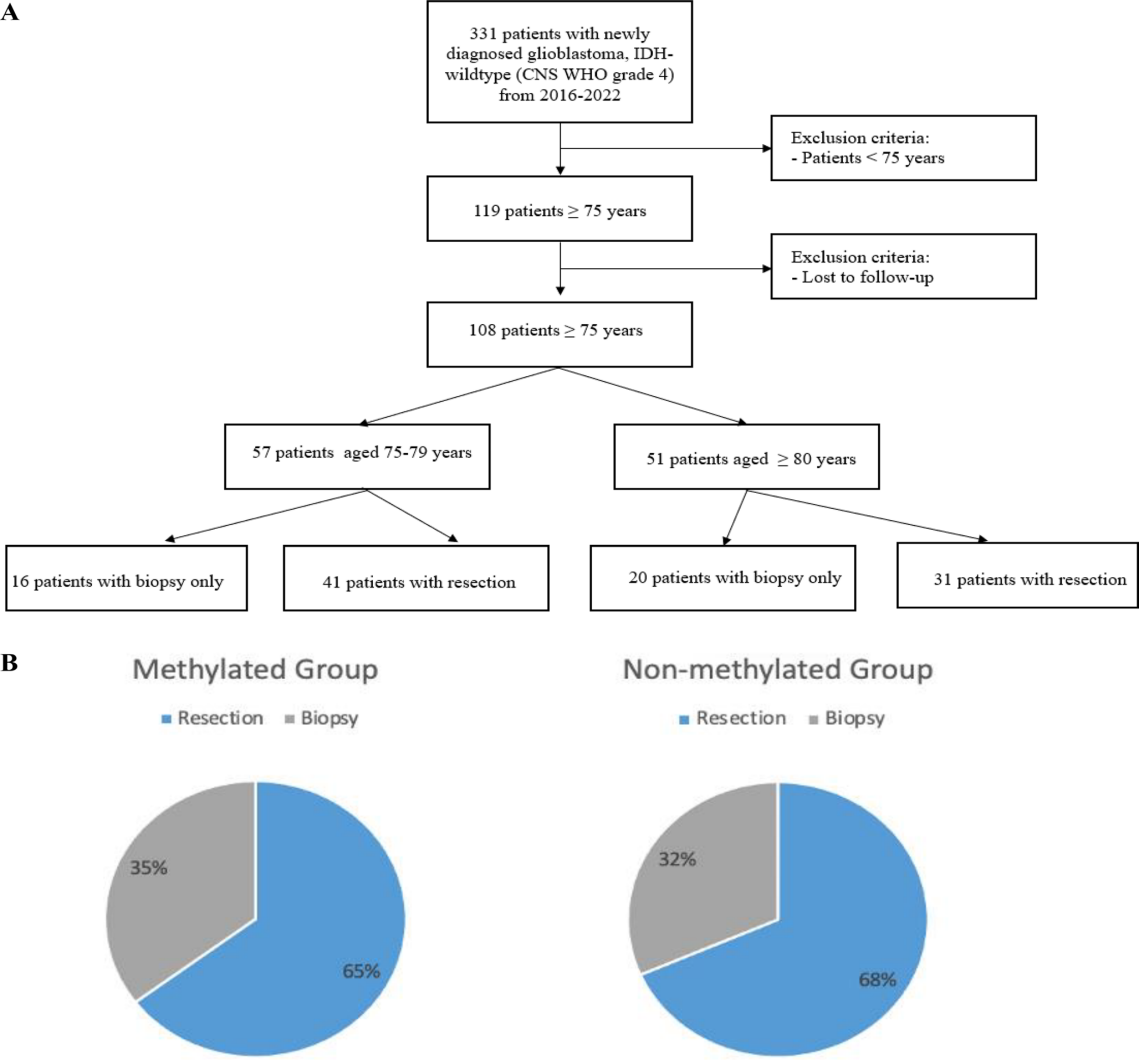
Flow chart of the patient cohort (**A**) and distribution of surgical strategy (resection vs. biopsy) according to MGMT promoter methylation status (**B**)

A total of 108 consecutive patients aged ≥ 75 years with newly diagnosed glioblastoma were included in the study (Fig. 1A). The cohort was stratified into two age groups: 57 patients aged 75–79 years (Group 1) and 51 patients aged 80 years older (Group 2)(Table 1). The median age in the overall cohort was 79 years (interquartile range [IQR]: 77–82). Females represented 46.3% of the total cohort, with a comparable distribution across age groups (43.9% in Group 1 vs. 49% in Group 2). The median Karnofsky Performance Status (KPS) at admission was 70 (IQR: 60–80) across all subgroups. The proportion of patients with MGMT promoter methylation was similar in both age groups (49.1% in Group 1 vs. 45.1% in Group 2), comprising 47.2% of the total cohort.

The majority of patients presented with at least one comorbidity. Cardiovascular comorbidities were the most frequent and were present in 58 patients (53.7%). Within this group, arterial hypertension was the most common condition, affecting 45 patients (41.7%), followed by other cardiovascular diseases, including coronary artery disease, atrial fibrillation, heart failure, or a history of myocardial infarction, observed in 32 patients (29.6%). Cerebrovascular comorbidities, such as previous stroke or transient ischemic attack, were present in 18 patients (16.7%). Metabolic comorbidities included diabetes mellitus in 19 patients (17.6%) and dyslipidemia in 30 patients (27.8%). Pulmonary diseases, including chronic obstructive pulmonary disease or chronic bronchitis, were noted in 13 patients (12.0%). A history of other malignancies was present in 14 patients (13.0%). Other comorbidities were observed in 27 patients (25.0%), while 9 patients (8.3%) had no relevant comorbidities at baseline. Some patients presented with more than one comorbidity.

Thirty-six patients (33.3%) underwent biopsy only, with a higher proportion in the older age group (28.1% in Group 1 vs. 39.2% in Group 2). The distribution of surgical strategy according to MGMT promoter methylation status is shown in Fig. 1B. In terms of resection, maximal resection was achieved in 42 patients (38.8%), while 30 patients (27.7%) underwent submaximal resection. The proportion of maximal resection was higher in patients aged 75–79 (45.6%) compared to those aged 80+ (31.4%). Overall, 21 of the 108 patients (19.4%) experienced postoperative complications.

Neurological complications included postoperative hemorrhage in five patients (4.6%), postoperative delirium in three patients (2.8%), new motor deficit in one patient (0.9%), postoperative aphasia in one patient (0.9%), hydrocephalus in one patient (0.9%), and seizure in one patient (0.9%). Infectious complications comprised pneumonia in four patients (3.7%), urinary tract infection in two patients (1.9%), meningitis in one patient (0.9%), and wound healing disorder in one patient (0.9%). Cardiovascular complications included pulmonary embolism in one patient (0.9%).

Regarding tumor-specific adjuvant therapy, radiochemotherapy was administered to 25.0% of the total cohort, while chemotherapy alone and radiotherapy were given to 20.4% and 15.7%, respectively. These distributions were similar across age groups. At tumor recurrence, 19 patients (17.6%) received further tumor-specific treatment. Of these, 14 patients were treated with chemotherapy alone, 2 with radiotherapy alone, and 3 with combined radiochemotherapy. Due to the small number of patients and the heterogeneity of post-recurrence treatment strategies, these therapies were not included in survival analyses.

**Table 1:** Demographic and clinical characteristics of the patient cohort

	Group 1 (Aged 75–79)	Group 2 (Aged 80+)	Whole cohort	*p*-value
N.o. patients, n	57	51	108	
Sex, female (%)	25 (43.9)	25 (49.0)	50 (46.3)	0.700
KPS at admission, median (IQR)	70 (60–80)	70 (60–80)	70 (60–80)	0.630
Methylated MGMT, n (%)	28 (49.1)	23 (45.1)	51 (47.2)	0.703
Biopsy only, n (%)	16 (28.1)	20 (39.2)	36 (33.3)	0.229
Overall survival (months), median (IQR)	4 (2–12)	3 (1–7)	4 (2–8)	0.212
Progression-free survival (months), median (IQR)	3 (2–5)	2 (1–5)	3 (1–5)	0.252
Maximal resection, n (%)	26 (45.6)	16 (31.4)	42 (38.8)	0.167
Submaximal resection, n (%)	15 (26.3)	15 (29.4)	30 (27.7)	0.830
Non-resective tumour-specific therapy, n (%)				
Chemotherapy only	13 (22.8)	9 (17.7)	22 (20.4)	0.634
Radiotherapy only	9 (15.8)	8 (15.7)	17 (15.7)	1.000
Radiochemotherapy	15 (26.3)	12 (23.5)	27 (25)	0.825

### Impact of surgical strategy on survival outcomes

**Fig. 2:**
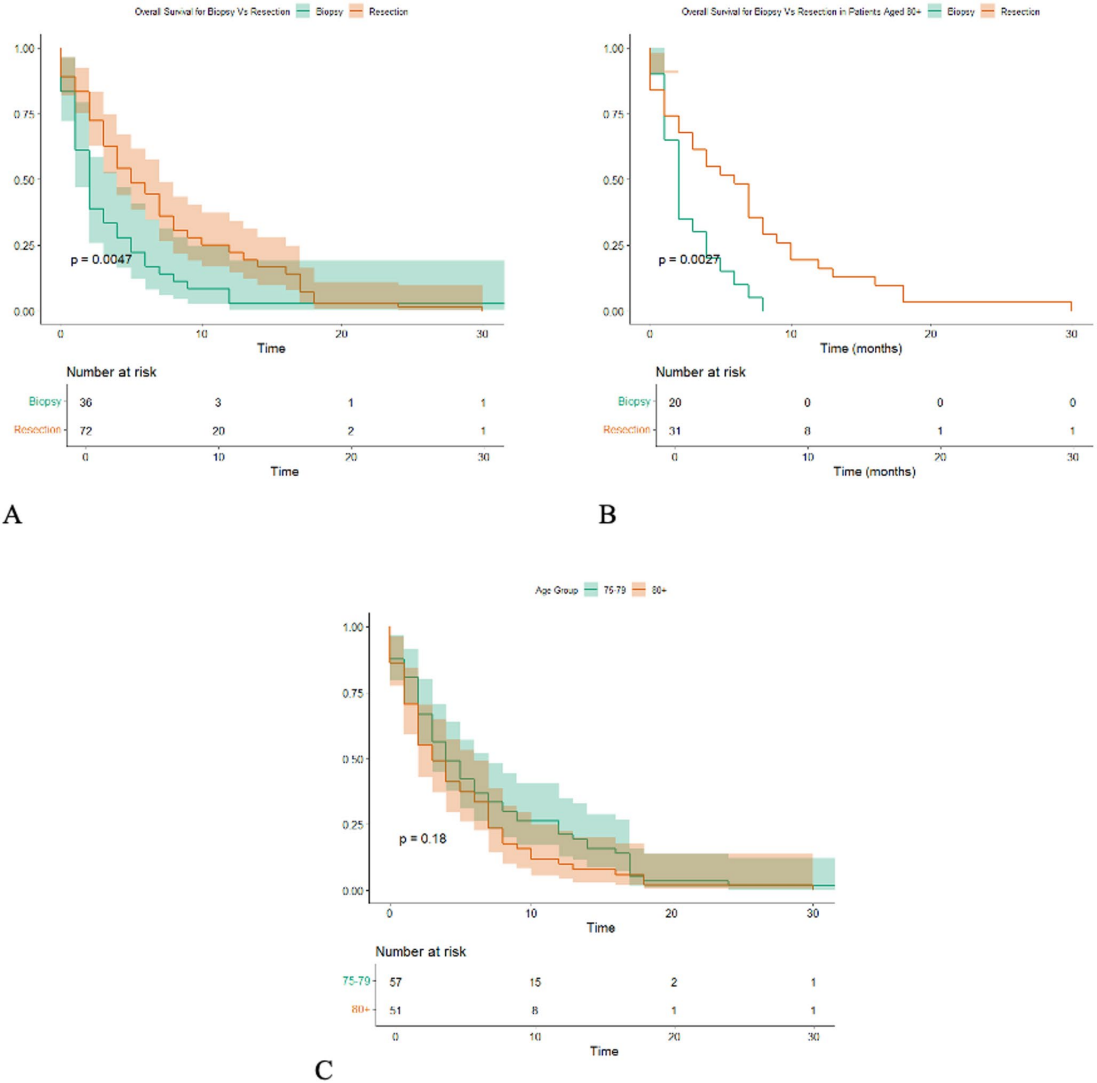
Overall survival stratified by surgical modality and age group. (**A**) Overall survival comparing resection vs. biopsy in the total cohort. (**B**) Overall survival in patients aged ≥ 80 years, stratified by surgical modality. (**C**) Overall survival stratified by age group (75–79, and ≥ 80 years)

In the total cohort, patients who underwent resection had significantly longer overall survival compared to those who received biopsy only (*p* = 0.0047; Fig. 2A). When stratifying by age, the survival advantage of resection remained statistically significant in patients aged ≥ 80 years (*p* = 0.0027, Fig. 2B). In contrast, overall survival did not differ significantly between the 75–79 and ≥ 80 age groups (*p* = 0.18; Fig. 2C).

**Fig. 3:**
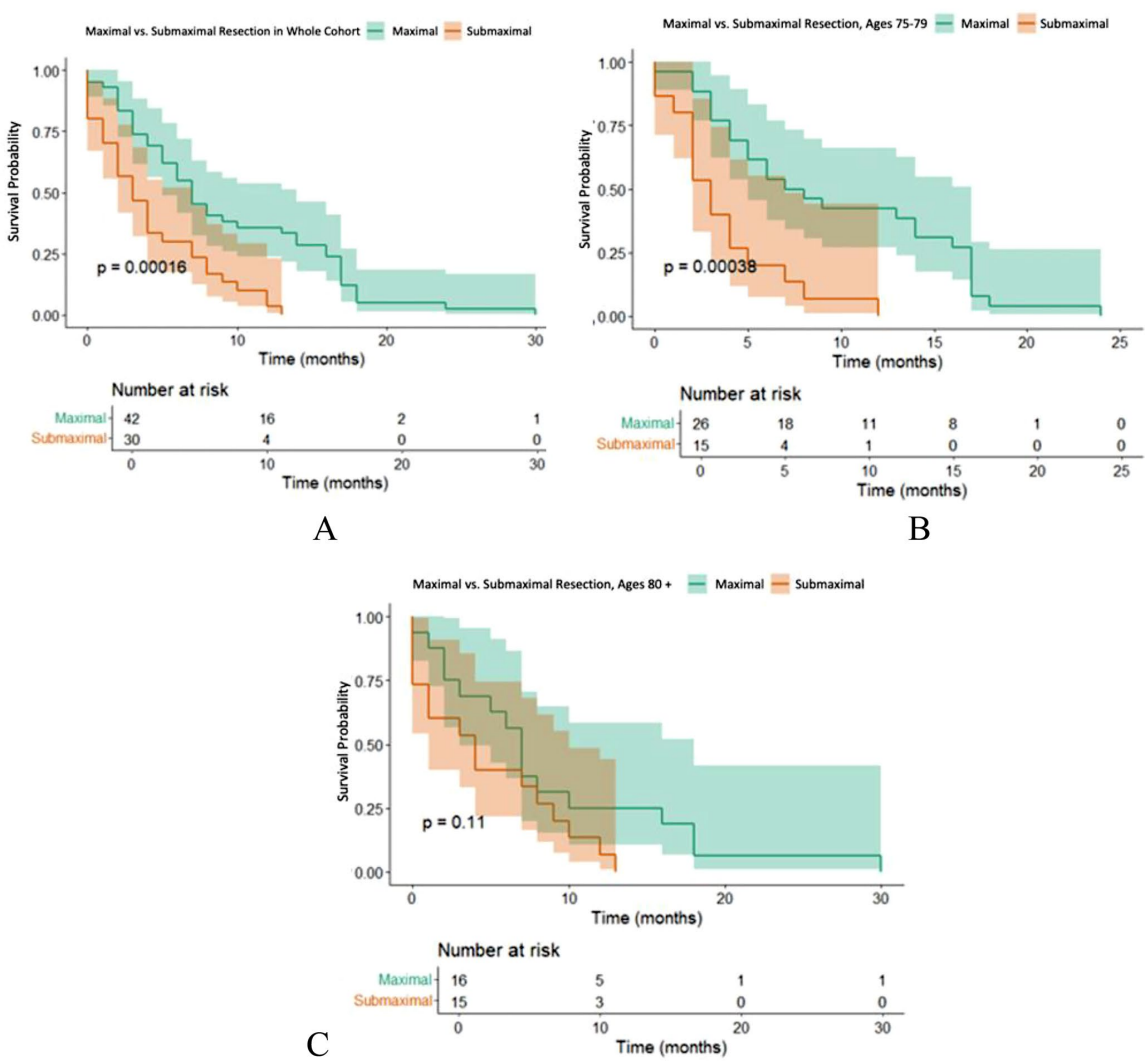
Overall survival stratified by extent of resection in the total cohort (**A**), aged 75–79 (**B**) and aged ≥ 80 years (**C**)

Maximal resection was significantly associated with improved overall survival compared to submaximal resection in the total cohort (*p* = 0.00016, Fig. 3A). In patients aged 75–79 years, maximal resection led to significantly longer OS compared to submaximal resection (*p* = 0.00038, Fig. 3B). In contrast, in patient aged ≥ 80 years, the OS benefit associated with maximal resection showed a favorable trend but did not reach statistical significance (*p* = 0.11, Fig. 3C).

### Prognostic impact of MGMT promoter methylation

**Fig. 4:**
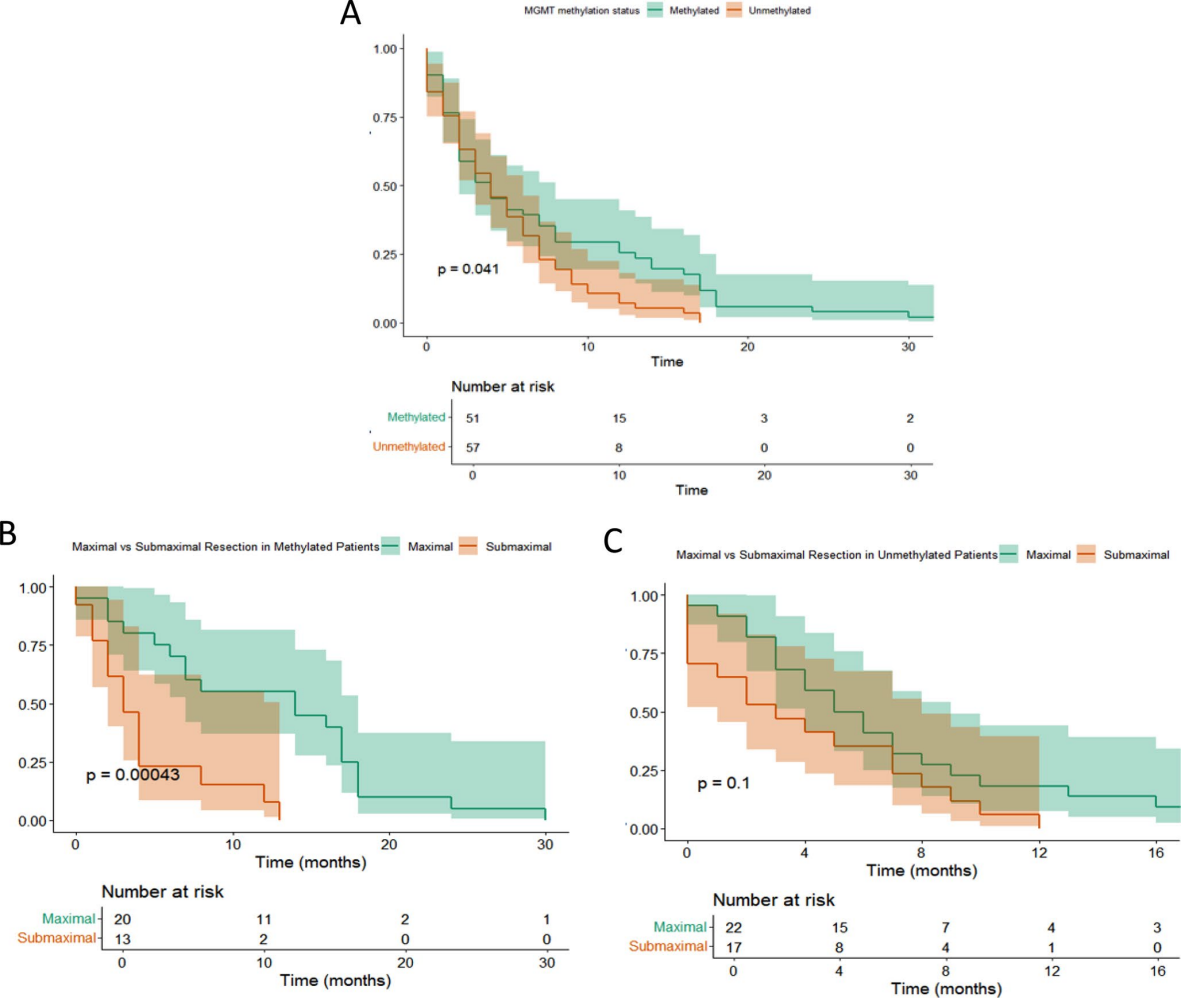
Overall survival stratified by MGMT promoter methylation status (**A**) and extent of resection in methylated (**B**) and unmethylated (**C**) patients

MGMT promoter methylation was significantly associated with improved overall survival (*p*=0.041; Figure 4A). When stratifying patients by extent of resection within MGMT subgroups, the benefit of maximal resection was clearly evident among patients with methylated tumors, who had significantly longer survival compared to those with submaximal resection (*p*=0.00043; Figure 4B). In contrast, among patients with unmethylated tumors, the difference in OS between maximal and submaximal resection did not reach statistical significance (*p*=0.1; Figure 4C), although a trend favoring maximal resection was still observed.

### Multivariate analysis

A multivariate Cox regression was performed to determine which factors independently influence overall survival (Table 2). Maximal resection remained an independent predictor of improved OS (HR 0.379, 95% CI 0.218–0.659, *p* < 0.001). Radiochemotherapy was associated with the strongest survival benefit among all treatment modalities (HR 0.174, 95% CI 0.0857–0.355, *p* < 0.001). Radiotherapy alone (HR 0.263, 95% CI 0.117–0.590, *p* < 0.001) and chemotherapy alone (HR 0.236, 95% CI 0.104–0.539, *p* < 0.001) also showed statistically significant associations with improved OS.

For progression-free survival (PFS), maximal resection significantly reduced the risk of disease progression compared to submaximal resection (HR 0.367, 95% CI 0.211–0.638, *p* < 0.001). Similarly, radiochemotherapy was associated with markedly improved PFS (HR 0.319, 95% CI 0.167–0.608, *p* < 0.001). Both radiotherapy alone (HR 0.301, 95% CI 0.140–0.650, *p* = 0.002) and chemotherapy alone (HR 0.280, 95% CI 0.130–0.604, *p* < 0.001) also showed significant associations with longer PFS.

**Table 2:** Multivariate analysis

Variable	Progression-free survival			Overall survival		
	HR	95% Cl	*p*-value	HR	95% Cl	*p*-value
Age	0.818	0.533–1.25	0.356	0.826	0.540–1.26	0.379
Biopsy	0.625	0.348–1.12	0.117	0.768	0.424–1.39	0.383
Maximal resection	0.367	0.211–0.638	**< 0.001**	0.379	0.218–0.659	**< 0.001**
Methylated MGMT	1.20	0.717–2.02	0.485	1.12	0.661–1.89	0.678
KPS at admission ≤ 70	0.729	0.435–1.22	0.230	0.886	0.517–1.52	0.659
Improved KPS at discharge	0.745	0.335–1.66	0.470	0.802	0.364–1.77	0.584
Radiochemotherapy vs. no treatment	0.319	0.167–0.608	**< 0.001**	0.174	0.085–0.355	**< 0.001**
Radiotherapy alone vs. no treatment	0.301	0.140–0.650	**< 0.001**	0.263	0.117–0.590	0.002
Chemotherapy alone vs. no treatment	0.280	0.130–0.604	**< 0.001**	0.236	0.104–0.539	**< 0.001**

HR: Hazard Ratio; 95% CI: 95% Confidence Interval

**Table 3:** Comparative analysis of published studies on glioblastoma in very elderly (> 75 years) patients

Study	*N*	Age Cutoff	Type of treatment	MGMT methylated	Surgical benefit
Baumgarten et al. [19]	143	≥ 75	Biopsy(72.7%), Resection(27.3%)	40%	yes, in univariate analysis only
Weller et al. [20]	76	≥ 80	Biopsy only	58%	n/a
Stadler et al. [21]	107	≥ 80	Biopsy(45%), STR(30%), GTR(25%)	33%	yes
Osawa et al. [22]	31	≥ 75	Biopsy (36%), Resection (64%)	76%	no
Present study	**108**	**≥ 75**	**Biopsy (33%)**,** Resection (67%)**	**47%**	**yes**

## Discussion

This study represents one of the largest single-center cohorts of patients aged over 75 years with newly diagnosed glioblastoma. When comparing the two surgical strategies — biopsy versus resection — we observed that patients who underwent surgical resection had longer overall survival compared with those managed with biopsy alone, and this survival advantage persisted even in patients ≥ 80 years. Our findings suggest that chronological age alone should not be considered an absolute contraindication to more aggressive resection, provided the patient is in good general condition and the procedure can be performed safely. These findings extend the current understanding of surgical benefit in the very elderly population, a group frequently underrepresented in clinical trials.

To our best knowledge, there is no generally agreed criterion for the definition of “older people” [19]. In certain developed counties, particularly in Western nations, the chronological age of 65 years is accepted as the definition of elderly. However, recent literature has shown that most geriatric symptoms, such as functional decline and severe hearing and vision problems generally appear between the ages of 70 and 75. By focusing on patients aged ≥ 75 years, our study addresses a group in whom therapeutic decision-making is often complicated by frailty, comorbidities, and limited life expectancy. Furthermore, the potential toxic side effect of tumor-specific therapies may outweigh the potential benefit for overall survival in this vulnerable group of patients.

According to the World Health Organization, the global population aged over 80 years is expected to triple by year 2025 [20]. A substantial proportion of individuals reaching this age will have a high quality of life along with preserved activity and functional levels [21]. Given the demographic changes and increased life expectancy, the geriatric population with glioblastoma is rapidly growing [22, 23]. Neverthless, the persistent underrepresentation of elderly patient in clinical trial has resulted in heterogeneous evidence regarding treatment efficacy in this growing patient group.

Traditionally, the treatment of newly diagnosed glioblastoma consists of surgery, radiotherapy and chemotherapy. The role of surgery in older patients with glioblastoma has been debated in recent publications (Table 3) [24–27]. 

In light of the heterogeneous and predominantly retrospective evidence, an international, multicentre prospective observational study (RESBIOP study) is currently recruiting patients and aims to compare biopsy versus resection in glioblastoma with respect to overall survival, functional outcomes, quality of life, and the ability to receive adjuvant therapy [28]. The results of this study may help to better define surgical decision-making, particularly in elderly patients.

Our results align with previous reports suggesting that surgical resection may be beneficial in carefully selected elderly patients, particularly those with preserved functional status as reflected by preoperative KPS. In this subset, survival outcomes may approach those observed in younger patients treated according to the standard of care.

Surgery should not be considered universally. In frail patients with poor functional reserve, large or multifocal tumors, or significant comorbidities, biopsy followed by supportive or palliative care may remain the most appropriate approach.

The landmark study by Stupp et al. demonstrated a significant survival benefit of radiotherapy with concomitant temozolomide followed by adjuvant temozolomide in patients with newly diagnosed glioblastoma [3]. However, this study included only patients younger than 70 years, thereby excluding a substantial proportion of the glioblastoma population. Subsequent RCTs addressed this gap [12–14]. Our findings reinforce these results: radiochemotherapy was the strongest survival benefit in patients ≥ 75 years followed by radiotherapy and chemotherapy alone, each of which provided an advantage over supportive care. Importantly, these results demonstrate that even in very elderly patients, adjuvant therapy should be considered, tailored to functional status and molecular profile.

MGMT promoter methylation is a well-established predictive biomarker for the response to alkylating chemotherapy [29–31]. Both NOA-08 and Nordic trial highlighted its particular importance in elderly patients, demonstrating that TMZ monotherapy is especially effective in those with MGMT promoter methylation. Consistent with prior literature, MGMT promoter methylation emerged as a strong prognostic factor in our cohort. Our study showed that the benefit of maximal resection was most pronounced among patients with methylated tumors, suggesting a synergistic effect between tumor debulking and subsequent alkylating chemotherapy. In contrast, among patients with unmethylated tumor, the difference in survival between maximal and submaximal resection did not reach statistical significance. Although surgical resection reduces the tumor mass, residual tumor cells have greater proliferative and infiltrative capacity, leading to early recurrence and limiting the long-term effect of extensive resection. In the unmethylated group, adjuvant chemotherapy had a limited effect, so maximal resection cannot be reinforced by effective systemic therapy.

Importantly, even in this very elderly subgroup, maximal resection was not associated with a higher rate of postoperative deterioration, which supports that in carefully selected patients, aggressive surgical strategies can be both feasible and safe.

This study has several limitations. First, it is a retrospective, single-center analysis, which inherently carries a risk of selection bias and limits the generalizability of the findings. Importantly, patients selected for biopsy-only differed systematically from those undergoing resection with respect to tumor location, extent, multifocality, performance status, and comorbidity burden. These baseline differences introduce substantial selection bias and preclude causal inference regarding the effect of surgical strategy on survival. Loss to follow-up represents an additional limitation of this study. Eleven patients were lost to follow-up, primarily due to transfer of care to other institutions, relocation, or incomplete follow-up information in the medical records, which is a known limitation of retrospective analyses. Standardized geriatric and frailty assessment was not performed; instead, functional status was evaluated using the Karnofsky Performance Status, which does not capture the full spectrum of frailty-related factors known to affect treatment tolerance and quality of life. In this analysis we did not analyse the side-effects of the specific tumor treatment regimens and their effects on neurocognition. Moreover, neurocognitive outcomes as well as frailty assessments were not available.

## Conclusion

In this retrospective cohort of glioblastoma patients aged ≥ 75 years, surgical resection was associated with longer overall survival and better functional outcomes compared with biopsy-only management. However, these findings should be interpreted cautiously, as patients selected for resection generally had more favorable baseline characteristics. Our results suggest that, in carefully selected elderly patients with good clinical condition, resection may be a reasonable and safe treatment option, particularly in the presence of MGMT promoter methylation.

## Data Availability

The datasets generated during and/or analysed during the current study are available from the corresponding author on reasonable request.
